# Vulval Aggressive Angiomyxoma in a 19 year teenager: a case report

**DOI:** 10.1186/s12905-022-01847-2

**Published:** 2022-09-19

**Authors:** Vitasta Muskan, Purbesh Adhikari, Baburam Dixit Thapa, Ramesh Shrestha

**Affiliations:** 1 B.P. Koirala Institute of Health Sciences (BPKIHS), Dharan, Nepal; 2 Department of Pathology, B.P. Koirala Institute of Health Sciences (BPKIHS), Dharan, Nepal; 3 Department of Obstetrics and Gynaecology, BPKIHS, Dharan, Nepal

**Keywords:** Aggressive Angiomyxoma, Case report, Histopathology, Radiological imaging, Wide local excision

## Abstract

**Background:**

Aggressive Angiomyxoma is a benign, slowly growing, locally aggressive and recurrent tumour that occurs in the pelvic-perineal region of females in their reproductive years. It presents as a painless, soft, gelatinous mass and metastasizes rarely. Suspicion can be made based on clinical examination and radiological imaging but final diagnosis is confirmed only after histopathology and immunohistochemistry. The choice of treatment is surgical wide local excision.

**Case presentation:**

We hereby present a case of a 19-year, unmarried lady, with a body mass index of 21 kg/m^2^, who presented with a chief complaint of painless mass in left vulva which progressively increased in size in the past one year. Clinical examination revealed a large, cauliflower like, exophytic mass of 10 cm × 10 cm size. Radiological imaging confirmed involvement of lymph nodes. Wide local excision with adequate tumour free margin and depth was used as a treatment modality. The diagnosis was confirmed via histopathological examination of the excised specimen. There is no recurrence in the patient up to date.

**Conclusion:**

Aggressive Angiomyxoma is a rare tumour and it is most often misdiagnosed. This report highlights the importance of considering Aggressive Angiomyxoma as a differential diagnosis of vulval masses and the two-step surgical approach for its treatment in low resource setting.

## Background

Aggressive Angiomyxoma (AA) is a rarely metastasizing, locally aggressive benign mesenchymal neoplasm, that presents as a painless mass involving the pelvic-perineal region of females of reproductive age [1–6]. The size ranges from 1 to 60 cm but, the chances of recurrence do not depend upon the size [4]. However, there are cases that have been reported in men and children, and involving uncommon sites like head, neck, lung, liver, urinary bladder, scrotum, inguinal area, spermatic cord and retroperitoneal regions [3, 4, 7–9]. In the latest World Health Organization classification, AA is now classified under tumours of uncertain differentiation. It must be distinguished from other more common benign and malignant myxoid tumours, cysts, hernia, abscess and lipomas [1, 8, 10]. AA was first mentioned in 1983 by Steeper and Rosai, after which about 150 cases have been reported up to date [1, 7, 8]. AA being a very rare tumour, is often misdiagnosed, the rates of misdiagnosis being 70–100% [1]. The diagnostic modalities which can be used are histopathological, radiological and immunohistochemical. However, other tumours may exhibit similarities in radiological imaging, so recognition must be based on histopathological and immunohistochemical findings [3]. The treatment of choice is surgical wide local excision, which prevents local recurrences [1, 5]. Local relapses often occur in about 30–40% of patients and may take many years for recurrence, so close monitoring is often required after surgery [1, 7].

## Case presentation

**Fig. 1:**
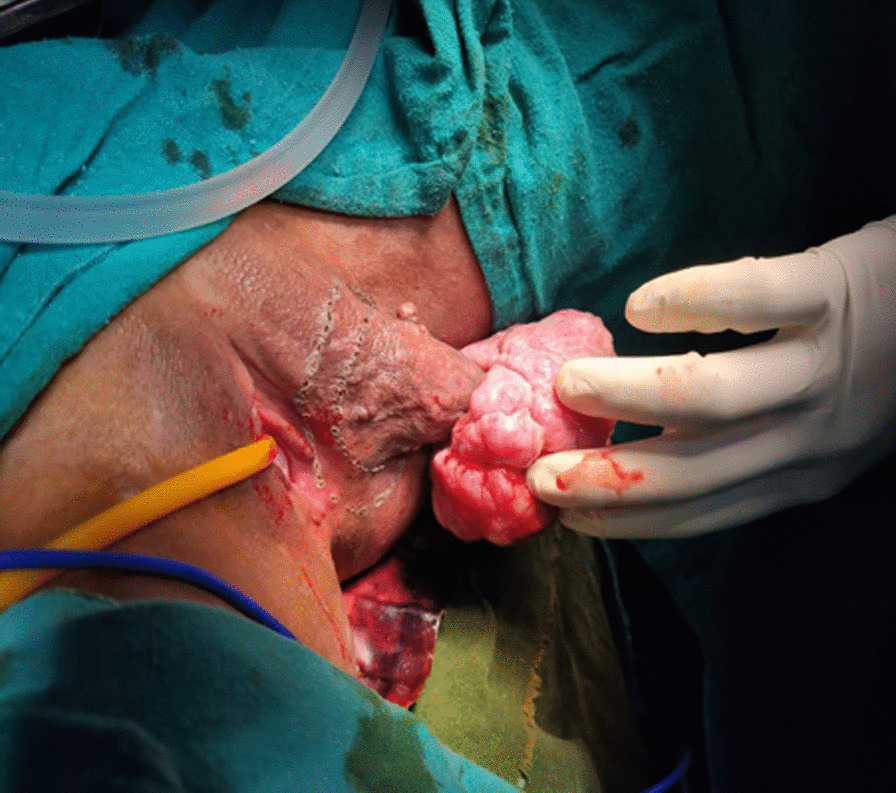
Preoperative image: mass in the left vulval region with irregular cauliflower like exophytic growth and resection margin of 1.5–2 cm all around the tumour (base of pedicle) margin made with pen cautery for wide local excision

A 19-year, unmarried lady from Dhankuta, Nepal with a body mass index of 21 kg/m^2^ presented to the Gynaecological Out-Patient Department on 12th August, 2020 with a chief complaint of painless mass in left vulva which progressively increased in size in the past one year. The vulval mass was associated with difficulty in ambulation. She had no any significant co-morbidities or family history of similar vulval masses. On examination, an irregular cauliflower-like single mass of 10 cm × 10 cm, was seen arising from the left vulval region on a thick pedicle (Fig. 1).

A Computed Tomography (CT) scan of whole abdomen and pelvis was done for radiological assessment of the nature of the mass which revealed, a large exophytic, lobulated, heterogenous mass, originating from the left vulval region, suspicious of malignancy. Along with the mass, multiple subcentrimetric discrete enhancing left external iliac and bilateral inguinal lymph nodes were reported, which raised the suspicion of metastasis in the lymph nodes.

She was admitted to the gynaecology ward with “vulval mass under evaluation” and a two-step surgical management plan was made due to unavailability of frozen section facility in our institute. In the first step the tumour was planned for wide local excision in view of excisional biopsy. In the second step, if the histopathological report was malignant then a revision surgery was planned to address the lymph nodes. So, after pre-operative counselling, detailed laboratory investigations and assessment of anaesthetic fitness was done, and surgery was performed on 19th August, 2020.

**Fig. 2:**
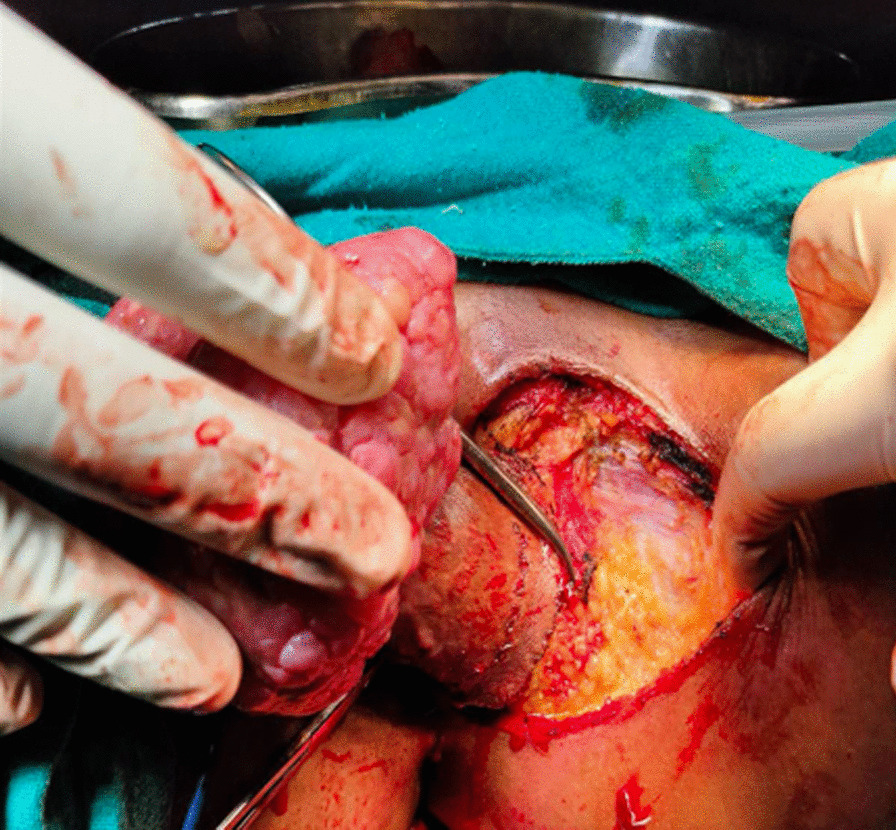
Intraoperative image: showing dissection deep up to the inferior layer of urogenital diaphragm to get adequate tumor free margin

A wide local excision was performed taking 1.5–2 cm tumour free margins all around and up to the inferior layers of the urogenital diaphragm under combined spinal and epidural analgesia (Fig. 2). The wound was approximated with delayed absorbable suture, total surgical blood loss was 200 ml, and total surgical duration was 90 min. Pre-operative antimicrobial prophylaxis was continued for 5 days in the postoperative period. Foley catheter was removed on the 3rd post-operative day and perineal wound care was done with daily sitz bath and pericare. Daily sitz bath was recommended using clean luke warm water in a clean tub under aseptic conditions for 5–10 min and pericare was done by cleaning the wound by normal saline, using a sterile gauze piece for dressing. This helps us to decrease the local inflammation and swelling in the wound and clean the debris along with some antibacterial properties. However, she developed perineal wound infection and wound dehiscence on 6th postoperative day and managed conservatively with daily wound dressing. She was discharged on 15th post-operative day. Hence the postoperative Clavein Dindo complication was Grade II.

**Fig. 3:**
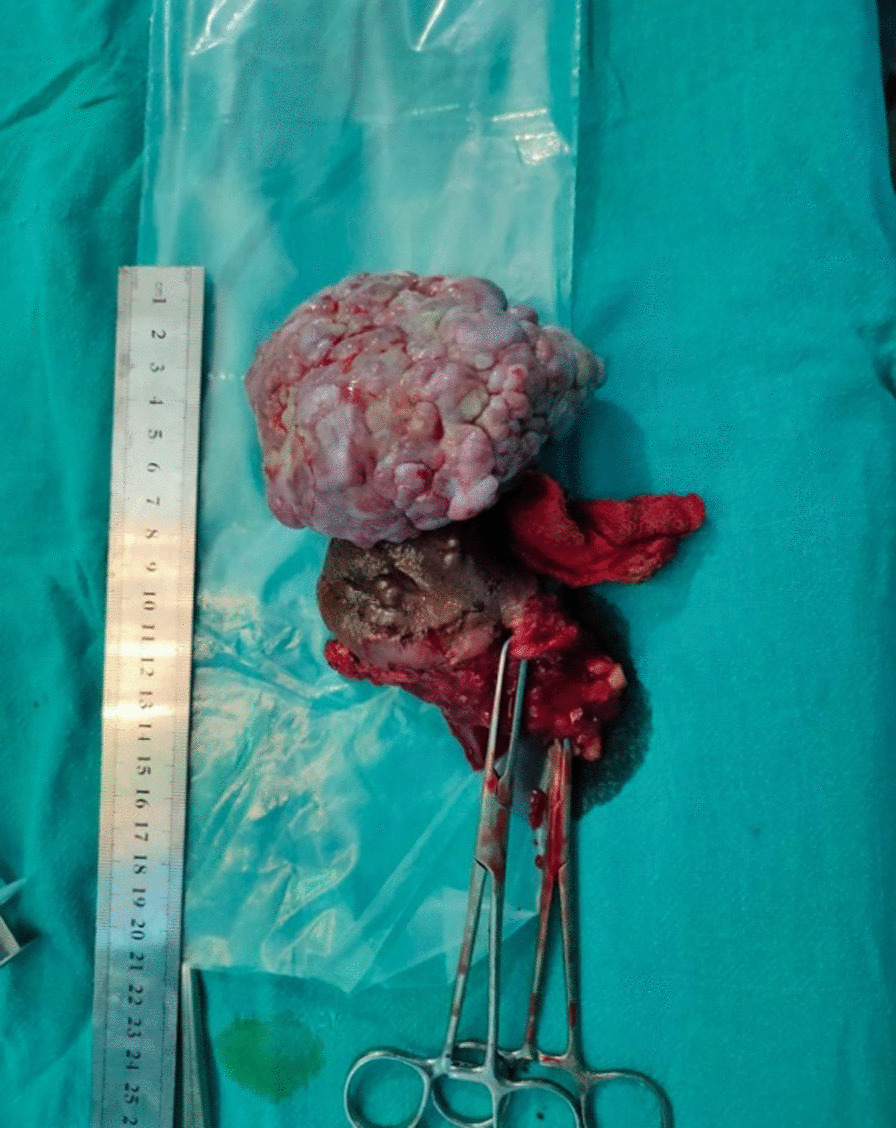
Post operative image: Excised specimen sized 10 cm × 8.5 cm × 8 cm showing irregular, cauliflower like growth for histopathological diagnosis

**Fig. 4:**
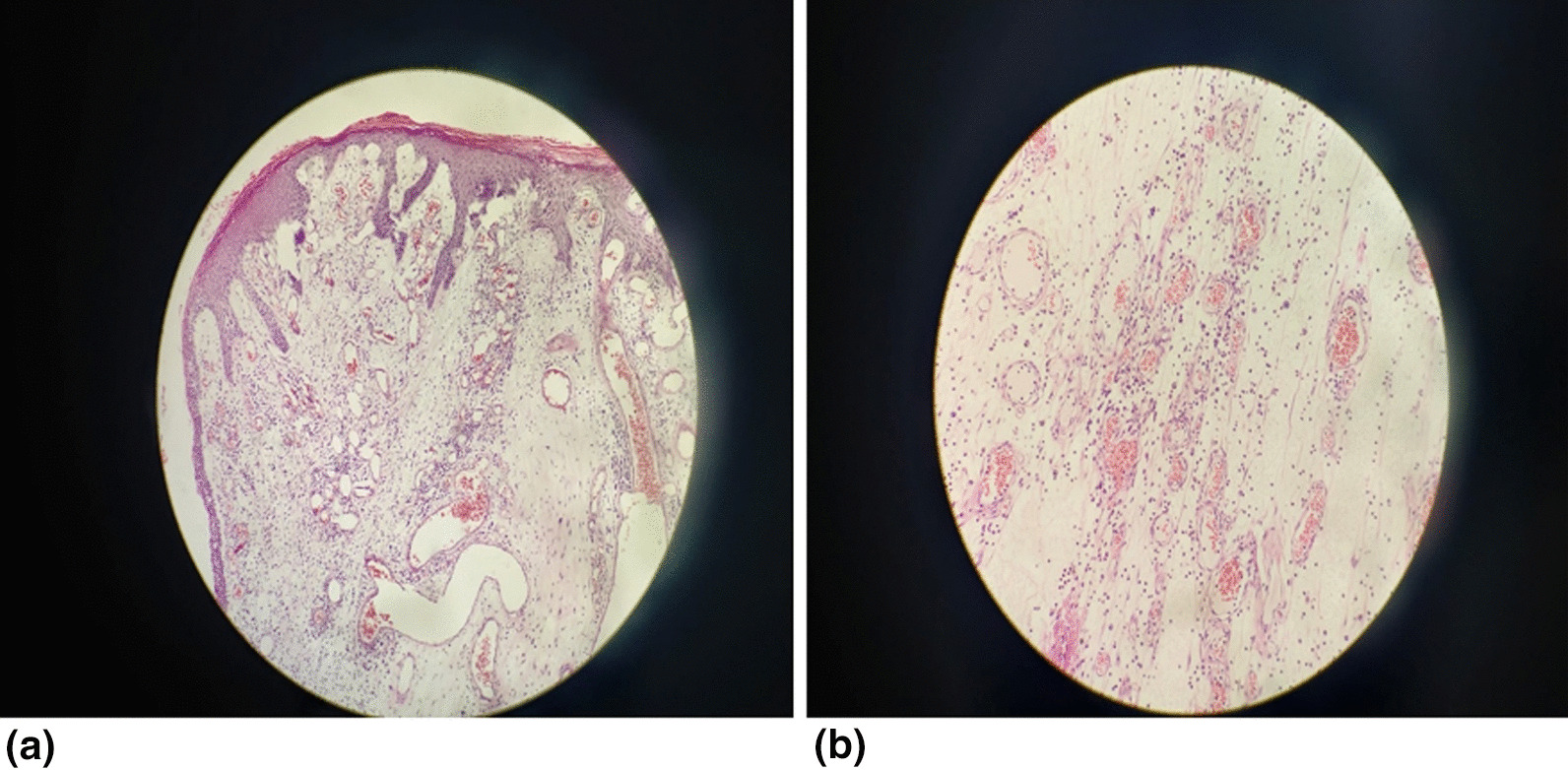
**a** Low power microscopic image: hypocellularity with prominent thin walled vascular channels in a myxoid stroma (H&E stain, × 100). **b** High power microscopic image: cytologically bland spindle cells are distributed evenly in the myxoid stroma with perivascular lymphocytes and scattered mast cells (H&E stain, × 400)

**Fig. 5:**
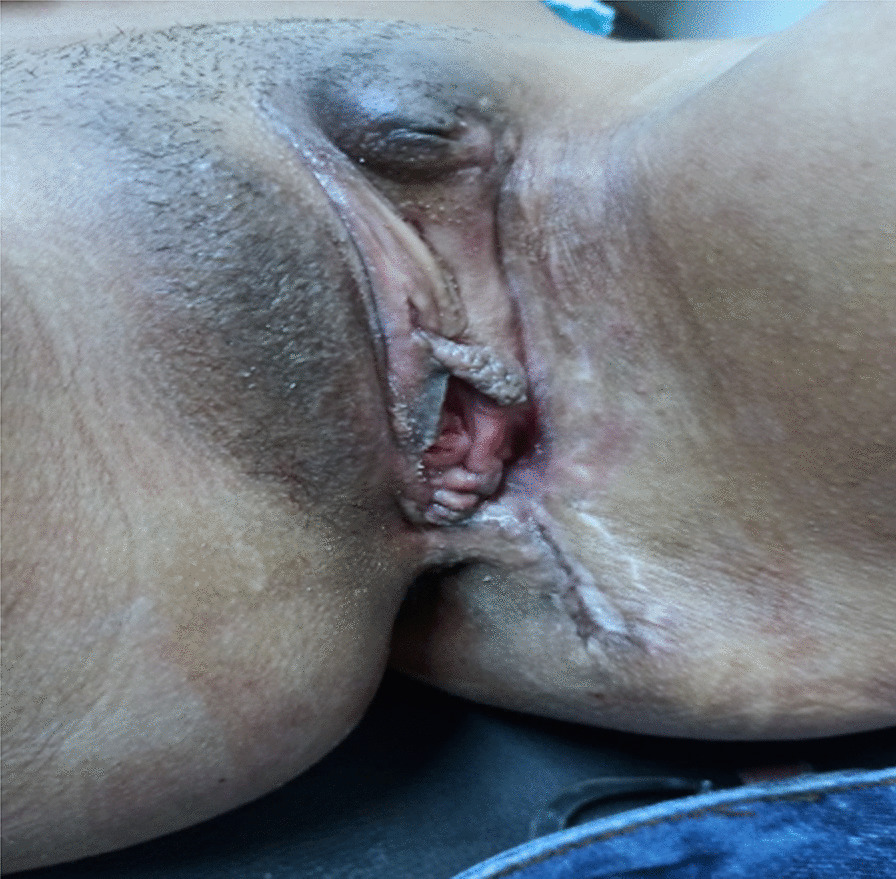
Follow up image: at 3 months of follow up showing healed scar and no recurrent lesions

The excised specimen was sent to pathology department for histopathological examination. On gross examination; the specimen was an irregular, pink to white coloured, friable tumour measuring 10 cm × 8.5 cm × 5 cm, 3 cm above the base extending from the skin surface (Fig. 3). The microscopic examination revealed tissue lined by keratinised squamous epithelium showing irregular acanthosis. Subepithelial region showed widely scattered proliferation of spindled to stellate shaped cells, with oval to spindle hyperchromatic nuclei, along with centrally to eccentrically placed nucleoli and eosinophilic to clear cytoplasm. Stroma showed myxoedematous matrix, containing varying number of prominent dilated thin to thick-walled blood vessels, along with collagen fibres, areas of haemorrhage, inflammatory cell infiltration comprising of lymphocytes, plasma cells and neutrophils. Few blood vessels were hyalinised with a prominent vascular smooth muscle. Nuclear atypia and atypical mitotic figures were not observed (Fig. 4a, b). All the resected margins were reported to be free of tumour cells. The diagnosis of AA was made. Fortunately, the histopathological reports showed a benign tumour with predominantly local invasion/ recurrence (AA) rather than lymphatic and hematogenous extension, so the revision surgery to address the lymph node was not done in our patient. However, she was planned for close observation in the post-treatment surveillance period. She is regularly followed up and there are no complications and clinical or radiological evidence of recurrence till date (Fig. 5).

## Discussion and conclusions

AA is a rare, locally aggressive, benign mesenchymal tumour usually seen in the pelvic-perineal region of females in their reproductive years [1–3, 8, 10, 11]. There are also cases reported in men and overall ratio of male:female is found to be 1:6 [10, 12]. The definite pathogenesis of the disease is not yet known, however, translocation of chromosome 12 has been implicated as a possible cause, which results in an aberrant expression of the high mobility group protein isoform I-C (HMGI-C) protein that is involved in DNA transcription [1, 3, 6, 8]. However, HMGI-C protein is also found positive in mesenchymal tumours other than AA [8]. As, the tumour is rare and has untypical symptoms, it is often misdiagnosed. The differential diagnosis of AA includes other myxoid tumours (angiomyofibroblastoma, fibroepithelial stromal polyp, myxomas, superficial angiomyxoma, myxoid neurofibroma, myxoid liposarcoma and myxofibrosarcoma), cysts (Bartholin’s, vaginal, labial, mesonephric duct), hernia (Vaginal, Levator ani muscle), abscess and lipoma [3, 5, 10, 11].

It is often presented as a slowly growing, painless mass, sometimes associated with bleeding (low haemoglobin levels), dyspareunia and change in bowel and bladder function [1, 2, 5, 6, 11]. In our case, the patient was a 19 year teenager who presented with slowly growing painless mass with difficulty in walking. Even though the tumour has a slow growing nature, she presented to us only when the tumour size was 10 cm, due to asymptomatic nature of growth in its initial presentation and far distance of our hospital from her location. In fact, she was reluctant for treatment and was brought to our hospital by her mother only when, she noticed the vulval mass in her daughter on enquiry of her altered gait. On examination, the mass was pedunculated, irregular, cauliflower like, which was in contrast to the case reported by Rezai et al. (Smooth, ball like, regular mass) and Ietto et al. (rubbery, poorly circumscribed, bulky, pink colour) [1].

The physical examination may not reveal the true extent of tumour and internally it may invade the adjacent organs, so the diagnosis should not depend upon the physical examination [6]. The diagnostic modalities of choice are radiological, histopathological and immunohistochemical assessment. Due to the gelatinous consistency of the tumour, the biopsy is often difficult to obtain however, the gold standard test still remains the histopathological test [6]. The tumour produces a characteristic appearance of swirled and layered tissue on both CT scan and Magnetic Resonance Imaging (MRI) but still, MRI is more specific and thus the study of choice [1, 2, 8, 10, 11]. Other imaging modality that can be used is Ultrasonography (transabdominal, transperineal, transvaginal) [8]. However, due to unavailability of MRI in our institute, we requested a CT scan. The swirled appearance was not seen in the CT-scan. On the contrary, similar to some reports, a lobular low-density mass and a progressive enhancement pattern was seen [10]. AA has been known to express oestrogen receptors (ER), progesterone receptors (PR), vimentin, desmin, and CD34, which may be the reason for its growth more quickly during pregnancy [1, 2, 8, 9, 11]. Some reports have shown negative S100 [2, 7, 9]. Xu et al. reported the growth of mass during pregnancy and no growth postpartum, with good maternal and child outcomes, but also reported a high risk of bleeding during pregnancy because of the abundant blood supply of the tumour [2]. However, some cases reported the growth of mass was higher in the females after giving birth rather than during pregnancy, which may be due to the masking effect of body weight on the soft tissues [7]. However, the patient in our case was not pregnant and had not given birth previously. Also, due to the unavailability of immunohistochemical tests, receptor positivity status could not be assessed in our case. Lymph node biopsy was not done because, bilateral inguinal lymph nodes were clinically not palpable and were subcentrimetric in size. Also, due to unavailability of frozen section facility in our institute, a two-step surgery was planned as mentioned earlier (based on the histopathological report) to address the suspicious lymph nodes. The discrete enhancing lymph nodes suspicious on radiological scan maybe reactive or infective. However, the patient has been also kept under strict surveillance with close observation for any enlarging lymph nodes or any local recurrence.

On gross examination of the tumour, it typically appears as tan pink to tan grey, uncapsulated, lobulated, solid and poorly demarcated mass with glistening smooth homogenous surface and some areas of necrosis or haemorrhage on the cut surface [1, 5, 11]. However in our case, the mass was reported to be irregular, friable, cauliflower like, pink to white in colour. Histologically, AA is paucicellular, poorly circumscribed tumour, composed of spindle shaped cells (round-to-ovoid nuclei with dispersed chromatin, eosinophilic cytoplasm) within a myxoid matrix with medium to large-sized, thick walled, hyalinized, vessels (which are surrounded by eosinophilic smooth muscle cells and fibrillar collagen) [3, 10, 11]. There no nuclear atypia and the tumour does not involve the surrounding tissues [3]. In our case the histopathological findings were similar, but along with it, inflammatory cells and areas of haemorrhages were also visible.

The treatment of choice for AA is wide surgical excision [1, 2]. However, the infiltrative pattern of the tumour into the parametrial and intraabdominal spaces, makes it indistinguishable from adjacent tissues [1]. Thus, achieving negative margins during surgery is often challenging [1]. Also, the lesions of AA, involving pelvic cavity or abdominal cavity is often larger, and its excision involves the removal of a part or all of the adjacent organs [5]. Some studies also report depression, irritability, insomnia, impaired body image, lack of energy, lack of sexual interest, and fertility related issues after surgery [6]. In a low resource setting like ours, due to unavailability of frozen section, a two- step management plan is an alternative approach as done in our case. However, in places where the frozen section facility is available, complete surgery would be done in the same setting based on the frozen section report. Only 3 cases of AA with distant metastasis have been observed worldwide which involved the lungs, lymph nodes, Inferior Venacava and right atrium [5]. In our case, there were enhancing lymph nodes reported in the CT scan, but still lymphadenectomy was not done in view of local infiltrating and local recurrent nature of the tumour rather than lymphatic route of spread as well as clinically non-palpable nodes. Additionally, pharmacological treatment with Gonadotrophin Releasing Hormone (GnRH) agonists, aromatase inhibitors, estrogen receptor (ER) or progesterone receptor (PR) blockers have been found effective [5]. Treatment with GnRH agonists have been found successful in cases where the tumour was hormone receptor positive and can be used pre-operatively to shrink the tumour (increases the chances of complete excision) [1]. However, in our case, as the margins were free from tumour cells and the patient was young (GnRH agonists have the ability to supress oestrogen production), GnRH agonists were not given to the patient. Attempts of treating the tumour by chemotherapy and radiotherapy have been unsuccessful, most likely due to the slow growth rate of the tumour [1, 4, 11]. However, some studies reported, the use of radiation therapy in recurrent lesions, prevented the recurrence for 2–3 years [5]. In our case, adjuvant chemotherapy and radiotherapy were not required as there was no recurrence after surgical excision. Our patient developed perineal wound infection on the 6th postoperative day, despite good perineal care because, 5th to 7th postoperative days is the usual time for wound breakdown, due to infection in majority of the patients. Also, as the site of surgery was near to the urethra and anal canal, the wound might have been infected by urinary and faecal microbes.

AA has a high rate of local recurrence, which can occur in decades, so it requires a long-term followup [1, 8, 11]. Initially, local recurrences were thought to be the results of inadequate primary resection and second wider resection was advocated, but it is not possible in large lesions [4]. Especially, lesions with positive margins are at a high risk of relapse due to the local infiltration capacity of the tumour [7]. However, a broad resection with tumour-free margins does not signify, that the recurrence will not occur and some studies suggest, equal recurrence rates in patients with positive and negative margins [4]. To prevent recurrence, techniques like angiographic embolization, beam irradiation and hormonal therapies (Tamoxifen, Raloxifene, GnRH agonists) are being used [1, 4]. However, some studies do not recommend the use of embolization, due to higher number of feeding vessels of the tumour [11]. The use of these techniques were not required in our case as there were no recurrences reported on follow-up.

AA is a rare, locally aggressive and recurrent, mesenchymal tumour with low metastasizing potential. It presents as a slowly growing painless mass in the pelvic-perineal region of females in the reproductive age group. Histopathology remains the gold standard tests for its diagnosis. Surgical wide local excision is the treatment modality of choice. Though AA is a rare tumour, treating it promptly, can prevent its local recurrences and invasion to local structures. This article highlights, the importance of considering AA as a differential diagnosis of exophytic/pedunculated large vulvar masses. As there are only 3 cases reported with involvement of lymph nodes, this article aids to challenge the classification of AA as a benign neoplasm and highlights the importance of further research in this case. Also, this article highlights about the two-step surgical approach based on the excisional biopsy report in low resource setting with unavailability of intraoperative frozen section.

## Data Availability

The datasets used during the current study are available from the corresponding author on reasonable request.
